# Clinical features of anemia in membranous nephropathy patients: a Chinese cohort study

**DOI:** 10.1080/0886022X.2022.2152692

**Published:** 2023-02-17

**Authors:** Zhe Li, Weibo Le, Haitao Zhang, Dacheng Chen, Wencui Chen, Shuhua Zhu, Ke Zuo

**Affiliations:** National Clinical Research Centre of Kidney Diseases, Jinling Hospital, Nanjing University School of Medicine, Nanjing, China

**Keywords:** Membranous nephropathy, anemia, cohort study

## Abstract

**Background:**

Anemia is a common complication in patients with progressive chronic kidney disease. This cohort study evaluated the prevalence, clinical features and prognosis of membranous nephropathy (MN) with anemia.

**Methods:**

We retrospectively analyzed a cohort of MN patients diagnosed using renal biopsy between February 2012 and February 2018. The clinical and pathological characteristics at baseline were recorded, and the outcomes (hemoglobin, proteinuria and renal function) during follow-ups were also evaluated. Univariate and multivariate logistic regression analyses were performed to identify the independent risk factors for anemia in MN patients. The MN patients were divided according to the therapeutic effect they experienced as follows: without-anemia, completely corrected anemia, standard anemia treatment and nonstandard anemia treatment groups. We compared the rate of complete remission of MN and renal end-point events among the four groups.

**Results:**

The median age of 483 patients was 42.43 (26.59, 50.20) years at the time of MN diagnosis. The prevalence of anemia at baseline was 23.81%, and the cumulative prevalence was 50.72%. There were 133 cases of mild anemia, 103 cases of moderate anemia and 9 cases of severe anemia; in addition, there were 228 cases of normocytic anemia and 17 cases of microcytic hypochromic anemia. Multivariate logistic regression indicated that acute renal tubule injury >5% (OR = 1.634, 95% CI 1.034, 2.581; *p* = 0.035), total protein level (OR = 0.949, 95% CI 0.923, 0.975; *p* < 0.001), cholesterol level (OR = 0.833, 95% CI 0.749, 0.926, *p* = 0.001), hypokalemia (OR = 2.612, 95% CI 1.227, 5.560, *p* = 0.013) and hypophosphatemia (OR = 2.653, 95% CI 1.303, 5.403, *p* = 0.007) were independent risk factors for anemia in MN patients. The complete remission rate of MN patients without anemia was significantly higher than that of anemia patients who exhibited treatment failure. The incidence of renal endpoint events was different among the four groups.

**Conclusion:**

The anemia experienced by MN patients is mainly mild and moderate, normocytic anemia. The pathological features of acute renal tubular injury and clinical nutritional status are independent risk factors for anemia. There were differences in renal prognosis among anemia patients with different treatment outcomes.

## Introduction

Anemia is the most common lesion of the blood system in patients with chronic kidney disease (CKD). Its severity is related to CKD and renal function. The incidence of anemia increases gradually with the decline in renal function [1]. The common causes of anemia in CKD patients include insufficient production and decreased activity of erythropoietin (EPO), lack of hematopoietic raw materials, hyperparathyroidism, shortened lifespan of red blood cells, and inflammation [2]. CKD patients with persistent anemia showed decreased quality of life and increased risk of cardiovascular disease and death [3]. Improving anemia in CKD patients can not only reduce the risk of cardiovascular disease but also delay the process of renal failure [4]. To date, studies on CKD with anemia have focused mainly on hemodialysis [5], peritoneal dialysis [6] and nondialysis chronic renal failure (CRF) [7]. There have also been cross-sectional studies on IgA nephropathy [8] and diabetic nephropathy [9]. Hemoconcentration due to hypoalbuminemia present in membranous nephropathy (MN) patients may mask the prevalence of anemia at clinical onset. There is a lack of cross-sectional studies in MN patients to report the prevalence of anemia at baseline and even fewer cohort studies to explore the cumulative prevalence of anemia during follow-up, as well as the efficacy and outcome of anemia on MN prognosis. In this study, we retrospectively analyzed a group of single-center observational MN cohorts to explore the prevalence, types, influencing factors and prognosis of anemia in MN patients to provide a reference for the prevention and treatment of anemia in MN patients.

## Materials and methods

### Patients

**Figure 1.**
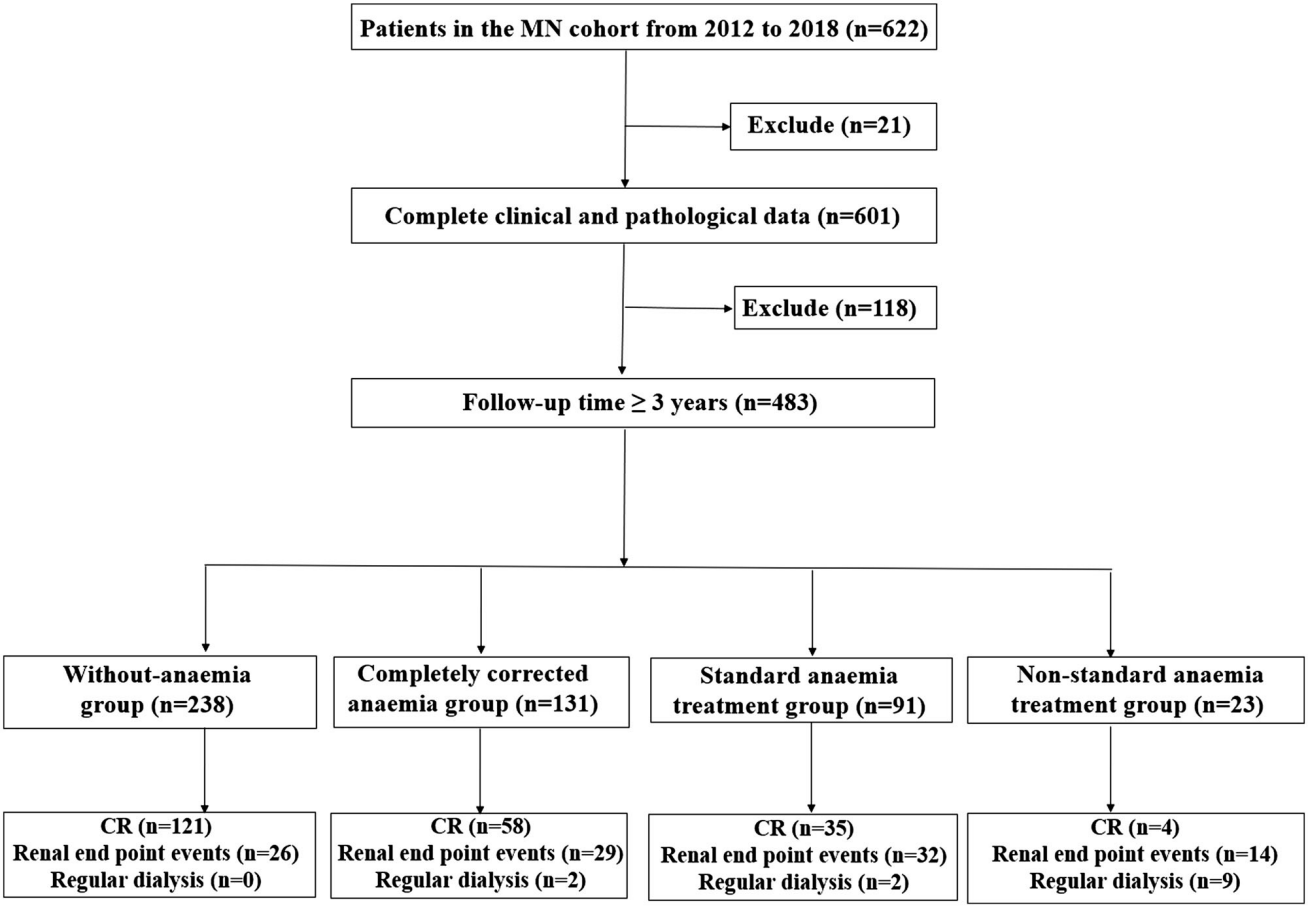
Screening process for the MN cohort. MN: membranous nephropathy; CR: complete remission.

Patients with MN diagnosed by renal biopsy in the National Clinical Research Center of Kidney Diseases, Jinling Hospital, Nanjing University School of Medicine, from February 2012 to February 2018 were selected as the subjects. All patients fulfilled the following criteria: (1) age ≥18; (2) glomerular pathology consistent with the light microscopy, electron microscopy and immunofluorescence characteristics of MN, and renal tissue sections were reviewed and confirmed by two pathologists; (3) within 3 months of the screening visit, the 24-h urine protein quantity was ≥0.5 g. Patients with any of the following conditions were excluded: (1) pregnant and lactating women; (2) hepatitis B and C virus infection, systemic lupus erythematosus, thyroid and other tumors, heavy metal and organic solvent poisoning and other clear secondary causes of MN; (3) glomerular proliferative lesions, mesangial areas and subcutaneous electron dense deposition found in renal pathology; (4) previous solid organ transplantation; (5) previous diagnosis of other kidney diseases; (6) complications with active bleeding, severe infection and other diseases that may lead to anemia; and (7) other severe systemic diseases that may endanger life within six months. The MN cohort study was approved by the Ethics Committee of Jinling Hospital (No. 2013KLY-012), and all the patients provided informed consent. A total of 622 patients were included in the MN cohort. In this study, 21 patients with incomplete clinical and pathological data and 118 patients with less than 3 years of follow-up were excluded, resulting in 483 patients being enrolled in this study (Figure 1).

### Clinical and laboratory parameters

The baseline data (such as demographic data, clinical manifestations, past history, medication history, physical examination, etc.) of the patients at the time of MN diagnosis and the results of relevant clinical laboratory tests (blood routine, blood biochemistry, immunology-related tests, urine routine, 24-h urine protein quantification, etc.) at the time of baseline enrollment were recorded. Patients were followed up once every 4 months during the first year and once every 6 months thereafter.

### Relevant diagnostic criteria and grouping

The diagnostic criteria for anemia were defined as hemoglobin (Hb) <130 g/L in males and Hb <120 g/L in nonpregnant females [10]. The criteria for mild anemia were 110 g/L ≤ Hb < lower limit of the normal reference value, those for moderate anemia were 80 g/L ≤ Hb <110 g/L, and those for severe anemia were Hb <80 g/L [11]. Mean corpuscular volume (MCV) <80 fL, mean corpuscular hemoglobin (MCH) level <27 pg and mean corpuscular hemoglobin concentration (MCHC) <320 g/L were required for microcytic hypochromic anemia. The criteria for normocytic anemia were 80 fL ≤ MCV ≤100 fL, 27 pg ≤ MCH ≤34 pg, and 320 g/L ≤ MCHC ≤360 g/L [12]. The criteria for complete correction of anemia were Hb ≥130 g/L in males and 120 g/L in nonpregnant females after anemia treatment. The criteria for standard anemia treatment were 110 g/L ≤ follow-up Hb < lower limit of the normal reference value. The criterion for nonstandard anemia treatment was follow-up Hb <110 g/L [10]. The criteria for complete remission (CR) of MN were 24-h urine protein content <0.3 g, serum albumin (Alb) >35 g/L and stable serum creatinine (sCr) for more than 6 months. The Chronic Kidney Disease Epidemiology Collaboration (CKD-EPI) equation was used to estimate the glomerular filtration rate (eGFR) [13]. Renal endpoint events were defined as eGFR reduction >30% within 2 years of follow-up or regular dialysis [14].

All patients underwent renal biopsy, and tissue samples were examined by conventional light microscopy, electron microscopy and immunofluorescence staining. The percentage of acute tubular injury (such as flattening of renal tubular epithelium, loss of brush borders) and renal interstitial fibrosis were observed by light microscopy. No related lesion was grade 0, lesion <5% was grade 1, 5% <lesion ≤25% was grade 2, 25% <lesion ≤50% was grade 3, and lesion >50% was grade 4.

### Statistical analysis

SPSS 19.0 statistical software was used for data processing. The measurement data with a normal distribution are expressed as the mean ± standard deviation, and the measurement data with a skewed distribution are expressed as the median (quartile 1, quartile 3). A *t* test or Mann–Whitney nonparametric test was used for comparisons between two groups depending on whether the data was normally distributed, and a univariate ANOVA test or Kruskal–Wallis *H* test was used for comparisons among four groups. Count data were expressed as percentages, and comparisons between groups were performed by *χ*^2^ test or Fisher’s exact test. A univariate logistic regression model was used to calculate the odds ratio (OR) of each factor, and variables with univariate *p* < 0.1 were included in the multivariate logistic regression model to further identify the independent risk factors for anemia. The CR rate and renal endpoint event rate were analyzed by the Kaplan–Meier method. *p* < 0.05 was considered statistically significant.

## Results

### Characteristics of the cohort

A total of 483 MN patients were included in this study, including 314 males (65.01%) and 169 females (34.99%). The median age of the MN patients at diagnosis was 42.43 (26.59, 50.20) years, and the positivity rate of blood anti-phospholipase A2 receptor (PLA2R) antibody detection was 63.15%. A total of 115 patients (23.81%) had baseline anemia at the time of MN diagnosis, 130 patients (26.92%) had anemia during the follow-up period, and the cumulative prevalence of anemia was 50.72%, including 133 cases (27.54%) of mild anemia, 103 cases (21.33%) of moderate anemia, and 9 cases (1.86%) of severe anemia. There were 228 cases of normocytic anemia (47.20%) and 17 cases of microcytic hypochromic anemia (3.52%). At the time of anemia, 179 patients had stage 1 CKD, 52 patients had stage 2 CKD, 13 patients had stage 3 CKD, and 1 patient had stage 4 CKD.

### Comparison of baseline anemia

This cohort was divided by anemia at the time of MN diagnosis. The levels of glomerular sclerosis, renal interstitial fibrosis, acute tubular injury, urinary N-acetyl-β-D-glucosaminidase (NAG), urinary retinol binding protein (RBP), blood C-reactive protein (CRP), hypokalemia, hypophosphatemia, and hypernatremia and the positive proportion of blood PLA2R antibody in MN patients with baseline anemia were higher than those in patients with baseline non-anemia (*p* < 0.05). In the baseline anemia group, the white blood cell (WBC) count, total lymphocyte count (TLC), total neutrophil count (TNC), red blood cell count (RBC), Hb, hematocrit (HCT), MCV, MCH, MCHC, red blood cell distribution width-coefficient of variation (RDW-CV), platelet (PLT) count, and total protein, Alb, globulin (Glb), prealbumin and cholesterol (Chol) levels were lower than those in the baseline non-anemia group (*p* < 0.05). There were no significant differences in sex, age, hypertension, diabetes, body mass index (BMI), urinary protein, sCr, blood cystatin C, blood urea nitrogen (BUN), uric acid (UA), triglyceride (Tg), corrected blood calcium or fasting blood glucose levels between the two groups (Table 1).

**Table 1.** Comparison of baseline anemia in MN patients.

Clinical variables	Total (*n* = 483)	Baseline non-anaemia group (*n* = 368)	Baseline anemia group (*n* = 115)	*p*-Value
Male, *n* (%)	314 (65.01）	242 (65.76)	72 (62.61)	0.536
Age (years)	42.43 (26.59, 50.20)	41.77 (26.57, 49.83)	44.78 (27.48, 51.83)	0.096
Glomerular sclerosis (%)	0.00 (0.00, 6.25)	0.00 (0.00, 5.71)	3.03 (0.00, 8.00)	0.012
Renal interstitial fibrosis, *n* (%)				0.047
Level 0	64 (13.25)	55 (14.95)	9 (7.83)	
Level 1	213 (44.10)	165 (44.84)	48 (41.74)	
Level 2	187 (38.72)	137 (37.23)	50 (43.48)	
Level 3	19 (3.93)	11 (2.99)	8 (6.96)	
Acute renal tubule injury, *n* (%)				0.008
Level 0	68 (14.08)	59 (16.03)	9 (7.83)	
Level 1	197 (40.79)	155 (42.12)	42 (36.52)	
Level 2	199 (41.20)	144 (39.13)	55 (47.83)	
Level 3	18 (3.73)	10 (2.72)	8 (6.96)	
Level 4	1 (0.21)	0 (0.00)	1 (0.87)	
Hypertension, *n* (%)	92 (19.05)	69 (18.75)	23 (20)	0.766
Type 2 diabetes, *n* (%)	32 (6.63)	22 (5.98)	10 (8.70)	0.306
BMI (kg/cm²)	23.65 (21.19, 26.04)	23.71 (21.13, 26.02)	23.50 (21.33, 26.31)	0.749
Urine test				
NAG (U/g*cr)	24.00 (14.50, 39.45)	23.40 (14.20, 38.35)	26.91 (15.70, 45.80)	0.025
RBP (mg/L)	0.40 (0.20, 1.00)	0.40 (0.20, 0.90)	0.50 (0.20, 1.70)	0.026
Urine protein (g/24h)	3.70 (1.95, 6.22)	3.63 (2.00, 6.21)	3.96 (1.76, 6.28)	0.775
Blood test				
CRP (mmol/L)	0.50 (0.10, 1.60)	0.50 (0.10, 1.50)	0.50 (0.10, 2.38)	0.011
WBC (×10^9/L)	8.35 (6.60, 11.00)	9.00 (7.00, 11.50)	7.30 (5.70, 9.20)	<0.001
TLC (×10^9/L)	2.26 (1.80, 3.03)	2.34 (1.85, 3.14)	2.05 (1.58, 2.61)	0.001
TNC (×10^9/L)	5.18 (3.78, 7.81)	5.54 (4.02, 8.26)	4.29 (3.18, 6.34)	<0.001
RBC (×10^12/L)	4.54 ± 0.55	4.73 ± 0.44	3.91 ± 0.36	<0.001
Hb (g/L)	136.0 (125.5, 148.0)	142.00 (133.00, 151.88)	115 (109, 122)	<0.001
HCT (L/L)	0.41 (0.38, 0.44)	0.43 (0.40, 0.45)	0.36 (0.33, 0.37)	<0.001
MCV (fL)	90.31 ± 4.57	90.64 ± 4.15	89.54 ± 5.67	0.040
MCH (pg)	30.10 (29.10, 31.25)	30.25 (29.30, 31.40)	29.60 (28.45, 30.80)	<0.001
MCHC (g/L)	334 (325, 343)	336 (326, 344)	330 (318, 337)	<0.001
RDW-CV (%)	13.05 (12.50, 13.60)	13.0 (12.5, 13.5)	13.20 (12.75, 13.80)	0.004
PLT (×10^9/L)	239.00 (197.00, 279.00)	242.00 (202.00, 279.00)	221.00 (181.50, 270.00)	0.028
Total protein (g/L)	50.61 ± 9.18	51.50 ± 9.19	47.78 ± 8.58	<0.001
Alb (g/L)	31.00 (26.55, 35.50)	31.95 (27.10, 36.38)	28.4 (24.6, 33.3)	<0.001
Glb (g/L)	19.05 (16.80, 21.60)	19.28 (17.06, 21.64)	18.30 (15.80, 21.25)	0.008
Prealbumin (mg/L)	338.14 ± 114.31	354.04 ± 110.29	285.67 ± 112.10	<0.001
Scr (mg/dL)	0.76 (0.62, 0.89)	0.76 (0.62, 0.88)	0.75 (0.61, 0.94)	0.888
BUN (mg/dL)	14.70 (11.20, 18.16)	14.65 (11.21, 18.08)	14.8 (11.2, 18.4)	0.813
UA (μmol/L)	355.72 ± 96.29	356.96 ± 94.13	351.78 ± 103.22	0.615
CystatinC (mg/L)	1.05 (0.88, 1.25)	1.04 (0.89, 1.22)	1.12 (0.87, 1.36)	0.163
Chol (mmol/L)	7.73 (6.39, 9.32)	7.88 (6.47, 9.58)	7.37 (6.17, 8.68)	0.042
Tg (mmol/L)	2.15 (1.57, 3.03)	2.15 (1.58, 3.03)	2.15 (1.53, 2.96)	0.937
K+<3.5 mmol/L, *n* (%)	36 (2.20)	22 (5.08)	14 (12.17)	0.027
Na+ >145mmol/L, *n* (%)	18 (3.73)	10 (2.72)	8 (6.96)	0.048
Ca2+<2.1 mmol/L, *n* (%)	302 (62.53)	215 (58.42)	87 (75.65)	0.001
Correction Ca2+<2.1 mmol/L, n (%)	58 (12.01)	41 (11.14)	17 (14.78)	0.294
P3+<0.96 mmol/L, *n* (%)	40 (8.28)	24 (6.52)	16 (13.91)	0.012
FBG (mmol/L)	5.12 (4.72, 5.71)	5.12 (4.65, 5.73)	5.13 (4.78, 5.66)	0.656
anti-PLA2R positive, *n* (%)	305 (63.15)	220 (59.78)	85 (73.91)	0.006

MN: membranous nephropathy; BMI: body mass index; NAG: N-acetyl-β-D-glucosaminidase; RBP: retinol binding protein; CRP: C-reactive protein; WBC: white blood cell; TLC: total lymphocyte count; TNC: total neutrophil count; RBC: red blood cell; Hb: hemoglobin; HCT: hematocrit; MCV: mean corpuscular volume; MCH: mean corpuscular hemoglobin; MCHC: mean corpuscular hemoglobin concentration; RDW-CV: red blood cell distribution width - coefficient of variation; PLT: platelet; Alb: albumin; Glb: globulin; Scr: serum creatinine; BUN: blood urea nitrogen; UA: uric acid; Chol: cholesterol; Tg: triglyceride; FBG: fasting blood glucose; anti-PLA2R: anti-phospholipase A2 receptor; Correction Ca2+ (mmol/L) = Ca2+(mmol/L)-0.025 × Alb (g/L)＋1.0(mmol/L).

### Risk factors for baseline anemia

Univariate logistic regression analysis showed that acute renal tubular injury >5%, total protein, Alb, Glb, prealbumin, and Chol levels, hypokalemia, hypophosphatemia and positive blood PLA2R antibody were risk factors for anemia in MN patients. The multivariate logistic regression model showed that acute renal tubular injury >5% (OR = 1.634, 95% CI 1.034, 2.581; *p* = 0.035), total protein level (OR = 0.949, 95% CI 0.923, 0.975; *p* < 0.001), Chol level (OR = 0.833, 95% CI 0.749, 0.926, *p* = 0.001), hypokalemia (OR = 2.612, 95% CI 1.227, 5.560, *p* = 0.013) and hypophosphatemia (OR = 2.653, 95% CI 1.303, 5.403, *p* = 0.007) were independent risk factors for anemia in MN patients (Table 2).

**Table 2.** Logistic regression analysis of risk factors affecting baseline anemia in MN patients.

Clinical variables	OR (95% CI)	*p*-Value	OR (95% CI)	*p*-Value
Acute renal tubule injur*y* > 5%	1.744 (1.143, 2.660)	0.010	1.634 (1.034, 2.581)	0.035
Total protein (g/L)	0.954 (0.931, 0.978)	<0.001	0.949 (0.923, 0.975)	<0.001
ALb (g/L)	0.934 (0.902, 0.966)	<0.001		
Glb (g/L)	0.918 (0.866, 0.974)	0.004		
Prealbumin (mg/L)	0.994 (0.992, 0.996)	<0.001		
Chol (mmol/L)	0.909 (0.829, 0.998)	0.045	0.833 (0.749, 0.926)	0.001
K+<3.5 mmol/L	2.180 (1.076, 4.416)	0.030	2.612 (1.227, 5.560)	0.013
P3+<0.96 mmol/L	2.316 (1.184, 4.531)	0.014	2.653 (1.303, 5.403)	0.007
anti-PLA2R positive, *n* (%)	1.906 (1.197, 3.036)	0.007	1.653 (0.999, 2.735)	0.050

MN: membranous nephropathy; Alb: albumin; Glb: globulin; Chol: cholesterol; anti-PLA2R: anti-phospholipase A2 receptor;.

### Follow-up

**Figure 2.**
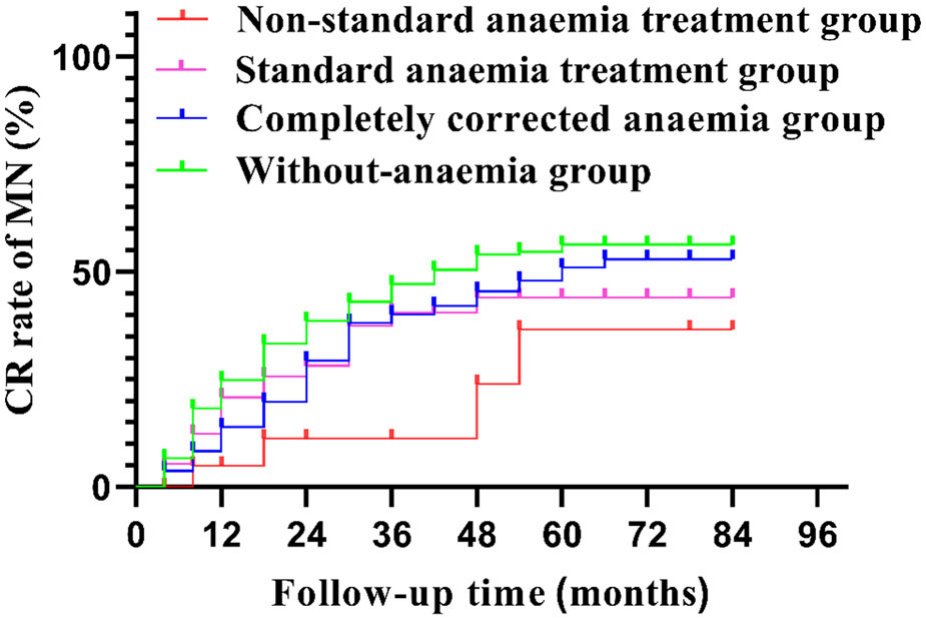
Comparison of CR curves of MN between groups in this cohort. MN: membranous nephropathy; CR: complete remission. CR rate: without-anemia group (50.84%) vs. nonstandard anemia treatment group (17.39%) (log rank test χ^2^=3.983; *p* = 0.046). There was no significant difference among the other groups.

**Figure 3.**
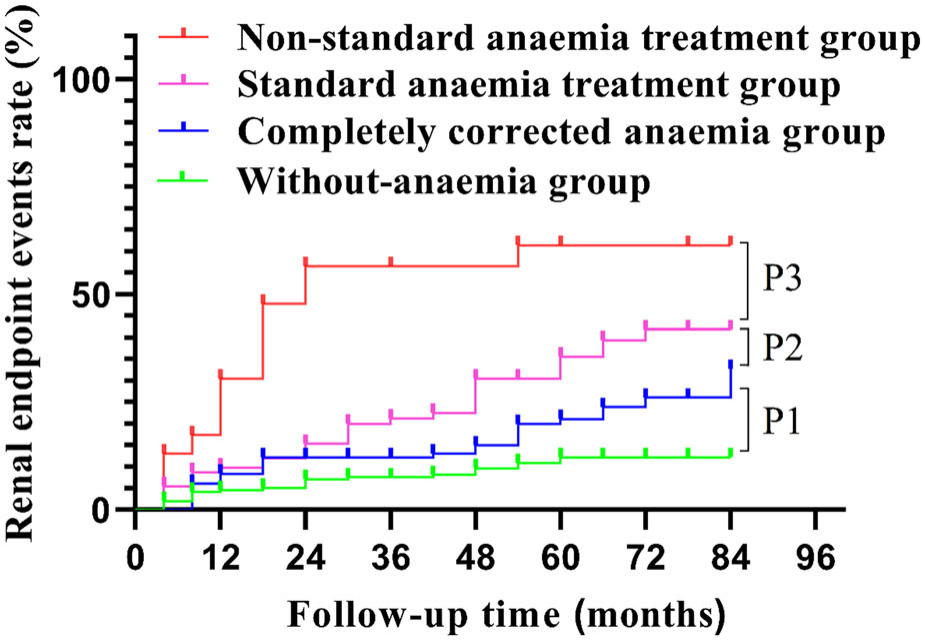
Comparison of renal endpoint event curves between groups in this study. The renal endpoint event rate was as follows: without-anemia group, 10.92%; completely corrected anemia group, 22.14%; standard anemia treatment group, 35.16%; and nonstandard anemia treatment group, 60.87%. There were significant differences among the groups (P1 = 0.006; P2 = 0.027; P3 = 0.005).

The median follow-up time was 60 (42, 72) months. Anemia patients were treated with oral iron and/or subcutaneous injection of erythropoiesis stimulating agents (ESAs), and the dosage was adjusted according to changes in Hb levels, with a maximum daily dose of ferrous succinate of 0.4 g and a maximum weekly dose of ESAs of 10,000 U. Two hundred and thirty-eight patients had no history of anemia. Anemia could be completely corrected in 131 patients. Ninety-one patients showed standard results, while for 23 cases, the anemia treatment results were not up to standard. Among anemia patients with different therapeutic outcomes, there were differences in levels of urine protein, NAG, RBP, blood WBC, TNC, RBC, Hb, HCT, MCH, MCHC, total protein, Alb, prealbumin, BUN, cystatin C at baseline and in the proportions of sex, anti-PLA2R positive, tacrolimus, cyclosporin A, cyclophosphamide, furosemide, ferrous succinate and ESAs (Table 3). A total of 218 patients (45.13%) were able to achieve CR, and the CR rate was 50.84% in the without-anemia group, 44.27% in the completely corrected anemia group, 38.46% in the standard anemia treatment group, and 17.39% in the nonstandard anemia treatment group. The CR rate in the without-anemia group was significantly higher than that in the nonstandard anemia treatment group (log rank test *χ*^2^=3.983; *p* = 0.046), and there was no significant difference among the other groups (Figure 2). Thirteen patients (2.69%) were treated with regular dialysis during follow-up, and 101 patients (20.91%) were followed up for 2 years with eGFR reduction >30%. The renal endpoint event rates were 10.92% in the without-anemia group, 22.14% in the completely corrected anemia group, 35.16% in the standard anemia treatment group, and 60.87% in the nonstandard anemia treatment group. There was no significant difference in the degree of renal interstitial fibrosis and acute renal tubular injury among the four groups when MN was diagnosed (Table 3), while there was a difference in the rate of renal endpoint events between the groups (*p* < 0.05) (Figure 3).

**Table 3.** Comparison of MN patient clinical characteristics with different therapeutic outcomes for anemia.

Clinical variables	Without-anemia group (*n* = 238)	Completely corrected anemia group (*n* = 131)	Standard anemia treatment group (*n* = 91)	Nonstandard anemia treatment group (*n* = 23)	p-value
Male, *n* (%)	165 (69.33)	94 (71.76)	42 (46.15)	13 (56.52)	<0.001
Age (years)	42.09 (28.04, 49.58)	42.43 (27.48, 51.20)	43.73 (25.60, 50.68)	34.18 (23.82, 51.63)	0.653
Glomerular sclerosis (%)	0.00 (0.00, 0.06)	0.02 (0.00, 0.06)	0.03 (0.00, 0.07)	0.04 (0.00, 0.10)	0.217
Renal interstitial fibrosis, *n* (%)					0.343
Level 0	28 (11.76)	18 (13.74)	16 (17.58)	2 (8.70)	
Level 1	117 (49.16)	57 (43.51)	32 (35.16)	7 (30.43)	
Level 2	86 (36.13)	50 (38.17)	39 (42.86)	12 (52.17)	
Level 3	7 (2.94)	6 (4.58)	4 (4.40)	2 (8.70)	
Acute renal tubule injury, *n* (%)					0.195
Level 0	31 (13.03)	17 (12.98)	18 (19.78)	2 (8.70)	
Level 1	107 (44.96)	49 (37.40)	33 (36.26)	8 (34.78)	
Level 2	95 (39.92)	57 (43.51)	37 (40.66)	10 (43.48)	
Level 3	5 (2.10)	7 (5.34)	3 (3.30)	3 (13.04)	
Level 4	0 (0.00)	1 (0.76)	0 (0.00)	0 (0.00)	
Hypertension, *n* (%)	45 (18.91)	23 (17.56)	21 (23.08)	3 (13.04)	
Type 2 diabetes, *n* (%)	11 (4.62)	17 (12.98)	3 (3.30)	1 (4.35)	
BMI (kg/cm²)	23.77 (21.29, 26.05)	23.63 (20.90, 26.40)	23.62 (21.01, 25.61)	23.51 (21.30, 25.95)	0.913
Urine test					
NAG (U/g*cr)	22.40 (13.10, 36.90)	27.45 (16.63, 44.23)	22.75 (14.50, 41.03)	24.10 (14.70, 58.70)	0.023
RBP (mg/L)	0.40 (0.20, 0.80)	0.60 (0.20, 1.65)	0.30 (0.20, 0.65)	0.55 (0.30, 1.10)	0.004
Urine protein (g/24h)	3.04 (1.78, 5.40)	4.28 (2.60, 7.29)	3.87 (1.79, 5.41)	5.03 (2.98, 8.55)	<0.001
Blood test					
CRP (mmol/L)	0.50 (0.10, 1.55)	0.50 (0.10, 1.75)	0.50 (0.10, 1.96)	0.30 (0.10, 0.78)	0.369
WBC (×10^9/L)	9.00 (7.09, 11.51)	8.10 (6.40, 11.00)	7.30 (6.00, 10.50)	9.30 (6.90, 11.50)	0.015
TLC (×10^9/L)	2.34 (1.84, 3.04)	2.23 (1.79, 3.15)	2.13 (1.76, 2.98)	1.91 (1.51, 3.34)	0.444
TNC (×10^9/L)	5.56 (4.11, 8.27)	5.01 (3.72, 7.44)	4.40 (3.50, 7.18)	5.81 (4.02, 7.71)	0.011
RBC (×10^12/L)	4.77 ± 0.43	4.33 ± 0.56	4.29 ± 0.55	4.30 ± 0.62	<0.001
Hb (g/L)	144.50 (135.38, 153.00)	129.00 (117.50, 141.50)	127.00 (116.00, 135.00)	123.00 (110.50, 136.00)	<0.001
HCT (L/L)	0.43 (0.41, 0.46)	0.39 (0.36, 0.43)	0.38 (0.35, 0.42)	0.39 (0.33, 0.43)	<0.001
MCV (fL)	90.52 ± 3.94	90.62 ± 4.56	89.55 ± 5.04	89.33 ± 7.69	0.200
MCH (pg)	30.50 (29.50, 31.46)	30.05 (29.10, 31.10)	29.60 (28.50, 30.70)	29.90 (28.00, 30.90)	<0.001
MCHC (g/L)	337.25 (328.00, 344.63)	333.00 (323.00, 340.50)	331.00 (320.50, 339.00)	329.00 (316.50, 336.00)	<0.001
RDW-CV (%)	13.00 (12.50, 13.60)	13.00 (12.50, 13.65)	13.10 (12.50, 13.50)	13.40 (12.85, 14.20)	0.176
PLT (×10^9/L)	241.75 (196.75, 280.50)	231.50 (197.00, 279.00)	231.00 (197.00, 263.00)	250.00 (199.00, 330.00)	0.487
Total protein (g/L)	52.74 ± 9.13	48.11 ± 8.93	49.50 ± 7.98	47.21 ± 10.53	<0.001
Alb (g/L)	32.83 (27.89, 37.40)	28.10 (24.80, 33.70)	29.50 (25.40, 34.80)	27.10 (24.60, 33.60)	<0.001
Glb (g/L)	19.30 (17.18, 21.70)	18.75 (16.40, 21.40)	18.80 (16.70, 21.40)	18.90 (14.60, 21.70)	0.342
Prealbumin (mg/L)	358.43 ± 112.38	322.77 ± 113.88	315.87 ± 111.04	300.18 ± 117.56	0.001
Scr (mg/dL)	0.76 (0.64, 0.87)	0.79 (0.66, 0.93)	0.69 (0.58, 0.85)	0.71 (0.58, 1.10)	0.113
BUN (mg/dL)	14.55 (11.19, 18.71)	16.15 (12.05, 18.40)	13.30 (10.80, 16.10)	14.75 (11.10, 20.40)	0.020
UA (μmol/L)	360.83 ± 96.04	355.10 ± 95.37	346.30 ± 94.54	343.74 ± 112.47	0.594
CystatinC (mg/L)	1.04 (0.91, 1.21)	1.11 (0.90, 1.29)	0.99 (0.81, 1.23)	1.18 (0.90, 1.41)	0.041
Chol (mmol/L)	7.51 (6.26, 9.12)	8.00 (6.68, 9.80)	7.62 (6.00, 9.22)	7.89 (6.89, 10.32)	0.118
Tg (mmol/L)	2.14 (1.61, 2.96)	2.16 (1.56, 3.27)	2.10 (1.30, 2.92)	2.06 (1.67, 3.68)	0.570
K+<3.5 mmol/L, *n* (%)	12 (5.04)	12 (9.16)	11 (12.09)	1 (4.35)	0.124
Na+ >145mmol/L, *n* (%)	9 (3.78)	4 (3.05)	5 (5.49)	0 (0.00)	0.737
Ca2+<2.1 mmol/L, *n* (%)	128 (53.78)	93 (70.99)	64 (70.33)	17 (73.91)	0.001
Correction Ca2+<2.1 mmol/L, *n* (%)	25 (10.50)	16 (12.21)	14 (15.38)	3 (13.04)	0.617
P3+<0.96 mmol/L, *n* (%)	20 (8.40)	9 (6.87)	9 (9.89)	2 (8.70)	0.835
FBG (mmol/L)	5.07 (4.61, 5.69)	5.26 (4.77, 5.73)	5.23 (4.81, 5.74)	4.83 (4.09, 5.91)	0.256
anti-PLA2R positive, *n* (%)	134 (56.30)	91 (69.47)	60 (65.93)	20 (86.96)	0.005
Drug					
Prednisone, *n* (%)	206 (86.55)	118 (90.08)	81 (89.01)	22 (95.66)	0.589
Tacrolimus, *n* (%)	89 (37.39)	68 (51.91)	43 (47.25)	15 (65.22)	0.007
Cyclosporin A, *n* (%)	21 (8.82)	24 (18.32)	20 (21.98)	6 (26.09)	0.002
Cyclophosphamide, *n* (%)	22 (9.24)	17 (12.98)	20 (21.98)	8 (34.78)	0.001
Rituximab, *n* (%)	1 (0.42)	2 (1.53)	0 (0.00)	0 (0.00)	0.394
ACEI/ARB, *n* (%)	198 (83.19)	111 (84.73)	76 (83.52)	15 (65.22)	0.175
Furosemide, *n* (%)	46 (19.33)	40 (30.53)	31 (34.07)	13 (56.52)	<0.001
Ferrous succinate, *n* (%)	0 (0.00)	55 (41.98)	52 (57.14)	23 (100.00)	<0.001
ESAs, *n* (%)	0 (0.00)	19 (14.59)	33 (36.26)	23 (100.00)	<0.001

MN: membranous nephropathy; BMI: body mass index; NAG: N-acetyl-β-D-glucosaminidase; RBP: retinol binding protein; CRP: C-reactive protein; WBC: white blood cell; TLC: total lymphocyte count; TNC: total neutrophil count; RBC: red blood cell; Hb: hemoglobin; HCT: hematocrit; MCV: mean corpuscular volume; MCH: mean corpuscular hemoglobin; MCHC: mean corpuscular hemoglobin concentration; RDW-CV: red blood cell distribution width - coefficient of variation; PLT: platelet; Alb: albumin; Glb: globulin; Scr: serum creatinine; BUN: blood urea nitrogen; UA: uric acid; Chol: cholesterol; Tg: triglyceride; FBG: fasting blood glucose; anti-PLA2R: anti-phospholipase A2 receptor; Correction Ca2+ (mmol/L) = Ca2+(mmol/L)-0.025 × Alb (g/L)＋1.0(mmol/L); ACEI: angiotensin converting enzyme inhibitor; ARB: angiotensin receptor blocker; ESAs: erythropoiesis stimulating agents.

### Analysis of not-standard anaemia treatment

Twenty-three patients did not reach the standard of anemia treatment during the follow-up. Compared with 222 patients whose anemia treatment was corrective or reached the standard, the proportion of microcytic hypochromic anemia was higher (21.74 vs. 5.41%; *p* = 0.014), and the Hb level [114(103, 120) g/L vs. 118(113, 124) g/L; *p* = 0.023] and MCV (87.62 ± 7.68 fL vs. 90.03 ± 4.90 fL; *p* = 0.036) for the first incidence of anemia were lower.

## Discussion

The anemia prevalence in CKD patients is higher than that in the general population [15]. The prevalence of anemia observed in a cross-sectional study of IgA nephropathy in Chinese adults was 21.3% [8]. In this study, it was found for the first time that the baseline anemia prevalence was 23.81% when MN was diagnosed, and the cumulative anemia prevalence during follow-up was 50.72%, comprising mainly mild and moderate normocytic anemia.

EPO is mainly synthesized by renal peritubular interstitial fibroblasts, and EPO levels rise rapidly under stimulation by blood loss or hypoxia. It was previously believed that chronic lesions in the renal tubules and interstitium lead to insufficient EPO production in chronic renal failure [2]. In this study, we found that acute renal tubular lesions at MN diagnosis were an independent risk factor for anemia. If there was no significant difference in baseline sCr level between the two groups, urine NAG and urine RBP levels in the anemia group were significantly increased, suggesting that some MN patients also have acute renal tubular injury when renal function is normal, which affects EPO secretion and leads to anemia.

A low-protein diet can delay the deterioration of renal function in CKD patients [16], but malnutrition caused by strict diet control is also a risk factor for increased mortality in hemodialysis patients [17]. In this study, low total protein levels, low Chol levels, hypokalemia and hypophosphatemia were found to be independent risk factors for anemia in MN patients. If there was no significant difference in urinary protein levels between the two groups, the total protein, Alb, Glb and prealbumin levels in the anemia group were all low, suggesting that some MN patients may have overcontrolled their protein intake, resulting in malnutrition and insufficient hematopoietic substances. Analysis of patients whose anemia treatment failed to reach the standard found that the proportion of microcytic hypochromic anemia was high, suggesting that malnutrition would also affect the therapeutic effect of anemia in MN patients.

MN patients had a relatively good prognosis, with spontaneous remission of proteinuria, stable renal function but persistent presence of urinary protein, and gradual deterioration of renal function accounting for 1/3 each [18]. Patients with renal tubular and interstitial injury had a poor prognosis [19]. This study found that the CR rate of MN patients without anemia was significantly higher than that of patients with nonstandard anemia treatment. Persistent unalleviated urinary protein damages renal tubules in MN patients and affects EPO production. Long-term use of tacrolimus, cyclosporine A [20], ACEI, ARB [21] in MN patients with persistent unalleviated urinary protein would also lead to chronic fibrosis of renal tubules and the interstitium or EPO resistance and would eventually make anemia in these MN patients difficult to treat.

Iron, vitamin B12 and folic acid deficiency are not only the cause of microcytic hypochromic anemia or megaloblastic anemia but also the main causes of poor therapeutic outcomes in patients on ESA therapy [22,23]. Oral iron can contribute to improving iron blood parameters and reducing the systemic inflammatory status in CKD patients [24]. Folic acid treatment attenuated oxidative/nitrosative stress and may be considered a possible strategy to improve redox status and anemia in CKD patients [25]. Therefore, the levels of serum ferritin, transferrin saturation, vitamin B12, and folic acid should be monitored regularly in CKD patients with anemia [10].

Current Kidney Disease: Improving Global Outcomes (KDIGO) clinical practice guidelines recommend that Hb ≥110–115 g/L be the target for anemia treatment in CKD patients, and it is strongly recommended that Hb >130 g/L be avoided [10]. In the CHOIR [26], TREAT [27] and Amgen studies [28], it was found that the risk of all-cause mortality, stroke, hypertension and vascular access thrombosis in CKD patients in the high-Hb group was significantly increased. However, the patients enrolled in the above studies were mainly diabetic nephropathy, hypertensive nephropathy and dialysis patients. In this study, it was found that the renal prognosis of MN patients without anemia was the best, that of the completely corrected anemia group was better than that of the standard anemia treatment group, and that of the nonstandard group anemia treatment was the worst. Anemia treatment needs to be explored in subsequent targeted clinical trials for MN and other chronic glomerulonephritis patients.

The limitation of our study is that the reticulocyte count, serum ferritin, transferrin saturation, vitamin B12, folic acid and other indicators of MN patients were not included in the analysis. The nutritional index was too singular, and bioelectrical impedance analysis was not used to evaluate the nutritional status. The follow-up time was insufficient, and the renal endpoint events were replaced by a > 30% decrease in eGFR within 2 years of follow-up to end-stage renal disease, which needs further study.

In summary, anemia is a common complication of MN patients, who exhibit mainly mild and moderate normocytic anemia. The pathological manifestations of acute renal tubular injury and clinical nutritional status are independent risk factors for anemia in MN patients. There were differences in the CR rate and renal prognosis in MN patients with different anemia correction conditions.

## Data Availability

All the data supporting our findings is contained within the manuscript.
